# The Other Side: How does Informed Choice Affect Induced Abortions among Reproductive-Age Immigrant Women in China—A Cross-Sectional Study

**DOI:** 10.3390/ijerph13101038

**Published:** 2016-10-24

**Authors:** Chuanning Yu, Junqing Wu, Yuyan Li, Ying Zhou, Rui Zhao, Honglei Ji, Yi-Ran Li, Ying Han, Qi Tong

**Affiliations:** 1 School of Public Health, Fudan University, Shanghai 200032, China; yu.chuanning@icloud.com (C.Y.); lyy1033@163.com (Y.L.); yingzhou2012@163.com (Y.Z.); zhaorui821030@163.com (R.Z.); hongleijish@163.com (H.J.); llyyyrr@sina.com (Y.-R.L.); 2 Key Lab of Reproduction Regulation of NPFPC, SIPPR, IRD, Fudan University, Shanghai 200032, China; 3 Guizhou Center for Disease Control and Prevention, Guiyang 550002, China; 4 Health and Family Planning Commission, Dong Cheng District, Beijing 100005, China; jessica-0623@hotmail.com; 5 Institute for Population and Family Planning, Chongqing 400000, China; sophie19910119@sina.cn

**Keywords:** induced abortion, informed choice, migrants, contraceptive methods, China

## Abstract

This study attempted to explore how informed choice on contraceptive methods influenced induced abortions among reproductive-age immigrant women in China. A total of 3230 participants were recruited in Beijing, Shanghai, and Chongqing. Information on informed choice was collected by questionnaires. The annual incidence rate (spells) of induced abortions was 0.46 (1500/3230) among the participants. The sequence from the highest score to the lowest was long-term, short-term and natural contraceptive methods (*p* < 0.0001). Significant differences of rates in induced abortions were found in region, occupation, length of the first immigration up to now (year), purpose for immigration, number of children, marital status, sex preference, contraceptive methods, deciders of contraceptive methods and side effects. In the zero-inflated negative binomial model, the joint impacts showed when a participant with one child employed condoms or family planning service providers as the deciders of contraceptive methods introduced intrauterine devices, the occurrence of induced abortions was more likely to be reduced. Women who underwent side effects using pills were more likely to have had induced abortions.

## 1. Introduction

It is estimated that approximately that from 1995 to 2008 one in five pregnancies worldwide ended in an induced abortion. In 2008, 86% of all induced abortions occurred in developing countries compared with 78% in 1995. In Asia, induced abortion rates ranged from 26 per 1000 in South Central Asia and Western Asia to 36 per 1000 in Southern Asia between 2003 and 2008 [1,2].

In China, there were 13 million induced abortions annually [3] by 2004. According to the 2014 China Health Statistical Yearbook, the number of induced abortions reached 9.17 million in 2008 [4]. A particular concern is that vast numbers of workers have been flooding into cities as the reform expanded and the economy flourished. By 2010, the immigrant population was 260.9 million, increasing from 11.6% in 2000 to 19.6% of the total population. Among the whole immigrant population, 47.5% were women [5], who were are increased risk of unintended pregnancies due to a younger reproductive age (between 30–34 years old), high mobilization, active sexual needs, unmet requirements for contraception and poor reproductive and contraceptive knowledge [6,7]. Then, as a probable consequence, induced abortions are inevitable. So far, some studies have shown that the prevalence of induced abortions among those immigrant women who had three pregnancies and gave no birth was higher than that of those who had the permanent residence registration [8].

The innovation of family planning policies has led to changes in fertility perspectives and contraceptive methods. Induced abortions are not mainly caused by policies either. Since informed choice on contraceptives was introduced to China, the pattern of single “birth control” has changed to be client-centered “multiple contraceptive choices”. Informed choice emphasizes that clients should select the method that best satisfies their personal and reproductive health needs, based on a thorough understanding of their contraceptive options. These options feature independence, instructiveness and multi-choice, rather than coerciveness, supervision and single choice before informed choice was implemented. As a crucial part of family planning policy, informed choice has contributed to the diversity of contraception, which not only makes women have different fertility desires, but also has an impact on induced abortions; for example, short-term and self-controlled contraceptive methods may lead to contraceptive failure due to inferior contraceptive effect, strong self-control and absence of supervision from service providers in term of condom use and the rhythm method. In January 2016, two-child policy was ultimately carried out against the decline of total fertility rate and the aging of population. With the loosened birth policy, women have more choices concerning giving birth and contraceptive methods. Informed choice is of more importance than ever before. Therefore, this study aims to analyze contraceptive knowledge, attitude and practice (K.A.P) on informed choice among reproductive age immigrant women, along with the insight into their effects on induced abortions.

## 2. Methods

### 2.1. Sampling Strategy and Study Population

The study was conducted in three cities and the study participants were recruited between August 2013 and August 2014 using a multistage sampling method. Three cities, including Beijing, Shanghai and Chongqing, with immigrant populations of 8.03 million, 10.00 million and 5.40 million, respectively, were selected. In each city, two districts were randomly selected. Quota sampling was adopted to recruit a composite sample which was approximately proportional to the whole immigrant population distribution at three types of location: (1) factories (manufacturing, electronics); (2) construction sites (building, decorating, and machining); (3) service sites (civil service, wholesale and retail trade, transportation, security, restaurants, hotels, administrative management, and individual operations). The inclusion criteria for respondents were: (1) rural-to-urban women; (2) aged from 20 to 49; (3) not registered as permanent residents in these three cities where they were working and living; (4) resided in the cities for at least half a year; (5) volunteered to participate in this study; (6) use of contraceptive methods; (7) not infertile according to their medical records. The field face-to-face investigation was carried out during June and August, 2014 with a final aggregate of 3988 reproductive immigrant women. To be specific, 1314 came from Beijing, 1743 came from Shanghai and came 931 from Chongqing.

#### Survey Methods

An anonymous self-administered questionnaire was pretested among 100 subjects. Their feedback was used to revise the questionnaires prior to the formal investigation. Participants were asked to fill out the questionnaire in an assembly room or some designated places near their workplace or living quarters. Beyond that, a well-trained investigator was available to assist participants when they encountered difficulties in filling out the questionnaires and missing data was checked at the end of the survey. The range of questions covered socio-demographic characteristics, immigration and obstetric histories, fertility desire and informed choice-related information.

### 2.2. Measures

#### 2.2.1. Social-Demographic and Obstetric Characteristics

The surveys included ten socio-demographics characteristics and two obstetric variables: (1) age (years), educational attainment, registered residence status, occupation, family per capita monthly income (in dollars), marital status, length of the first immigration up to now (years), length of staying in the city per year (months), purpose for immigration and whether having medical insurance or not in the city; (2) sex preference and number of children. Sex preference was defined as the difference of number of sons expected minus the number of daughters expected. This could be grouped into three categories: “son preference” (the difference is more than 0), “no sex preference” (the difference is 0) and “daughter preference” (the difference is less than 0).

#### 2.2.2. Informed Choice-Related Knowledge

Knowledge on contraceptive methods consisted of three parts, which included short-term contraceptive methods (condom and pill), long-term contraceptive methods (IUD and tubal sterilization), and natural contraceptive methods (withdraw and rhythm method). Each method contains five aspects: awareness, usage, rationale, advantages, and side effects. For awareness, using one method is given one score. For the other four aspects, each of them has different answers and only one answer is correct. Meanwhile, it was credited with one score. For example, there are seven answers to the rationale for condom use, while only one out of the seven (stopping sperm from entering the vagina as a barrier function) is true. As a result, the overall score of each part is the sum of the correct answer scores of five aspects. Then, it was converted to centesimal system as scores of contraceptive methods in the final analysis. The answer to the question “which period is correct for female ovulation?” is evaluated by five items: 4–6 day before the next menstruation, the 14–16 day before the next menstruation, the 14–16 day after the end of menstruation, the 4–6 day after the end of menstruation, and unknown.

#### 2.2.3. Attitudes and Behavior of Informed Choice

Contraceptive methods expected to use and in use were grouped into five categories, including “IUD”, “condom”, “pill”, “tubal sterilization” and “other (withdraw/rhythm method or Norplant)”. The expected deciders of contraceptive methods could be defined as husband/sexual partner, wife/sexual partner, and both husbands and wives/sexual partners. Additionally, the deciders of contraceptive methods have three options: couples/sexual partners, family planning service providers (FPSPs) and physicians/community health workers. According to the fact whether they occurred or not, the side effects were classified as “yes” and “no”. Regarding the attitudes and behaviors of RHS, the participants were asked whether they agreed that the men should receive reproductive health education or not and whether the participants received (RHS) or not respectively. The response options were either “yes” or “no”.

### 2.3. Model Selection

**Figure 1:**
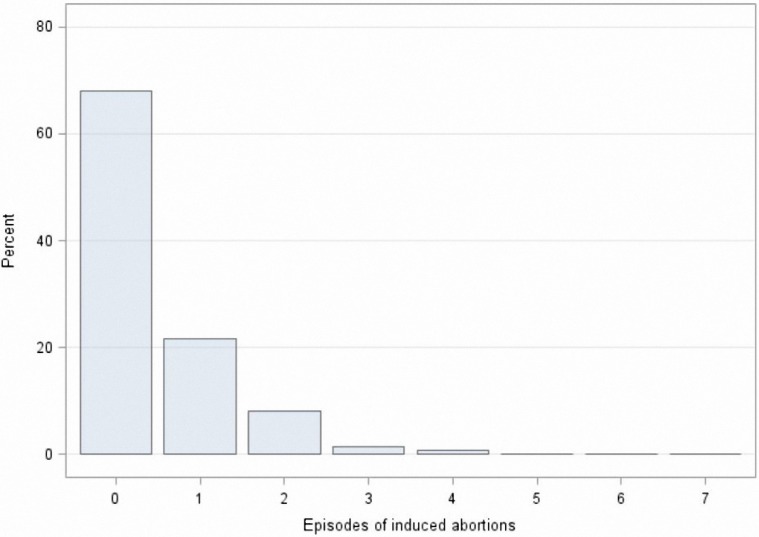
Distribution of induced abortions among the studied reproductive-age migrant women.

The researchers firstly took the zero-inflated negative binomial (ZINB) models into consideration because of the independent variable features that the percentages of induced abortion episodes for zero and one were 67.93% and 21.52%, respectively. Additionally, the mean and variance were 0.46 and 0.65, respectively (Figure 1). The Vuong test [9] for comparing non-nested models reflected that the ZINB model could better fit these data than the NB model.

### 2.4. Ethical Approval

Before the implementation of the research, the study protocol was approved by the Research Ethics Committee of the Shanghai Institute of Planned Parenthood Research (code: PJ2014-20). The purpose of the study had been interpreted to all the eligible participants. Meanwhile, verbal and written informed consents were obtained for information security and privacy protection. Notably, the consents were obtained from all the participants prior to the data collection.

## 3. Results

### 3.1. Socio-Demographic Characteristics and Obstetric History

According to this study, 3230 participants who met the inclusion criteria were included in the further analysis. The number of induced abortions, including single and repeated abortions, was 1036, and the annual incidence rate of induced abortions was 0.46 (1500/3230). Generally speaking, more than 90% of the participants were married and most were 20–39 years old, and over 60% of the respondents had rural residence registrations. Approximately 40% of them had graduated from junior high school and over 45% of them reported that it had been 11 or more years since their 1st immigration. Moreover, each year, the majority (94.64%) of them had stayed in the destination cities for at least 10 months. Three-quarter of the participants migrated for the purpose of work, and more than half of them were service workers. The respondents who had medical insurance exceeded half of the total. Most immigrant women (63.16%) had one child, and 28.14% of them had two children. Very few participants (3.90%) expected to have more than two children. The proportion of “no sex preference” accounted for 80.34%. The socio-demographic characteristics of the study population are summarized in Table 1.

### 3.2. Knowledge of and Attitudes to Induced Abortions Related to Informed Choice

The score medians were 20 (Inter-Quartile Range, IQR: 40), 20 (IQR: 50) and 0 (IQR: 40) in terms of short-term, long-term and natural contraceptive methods, respectively. As shown by the Wilcoxon signed rank test, the order from the highest score to the lowest was long-term, short-term and natural contraceptive methods (the long-term > the short-term: S = 319,818, *p* < 0.0001; the short-term > the natural: S = 329,877.5, *p* < 0.0001; the long-term > the natural: S = 225,384.5, *p* < 0.0001). Less than one-third of participants (28.61%) could correctly identify the female ovulation period.

Around two fifths (40.50%) and over one third (34.92%) of the participants were inclined to adopt IUD and condoms, respectively, as the contraceptive methods they expected to use. The first concern when choosing a contraceptive method was the effectiveness (83.96%), and the second was the convenience of use (41.64%). More than two fifths (43.00%) of participants thought that both husbands and wives/sexual partners should determine the contraceptive methods. Beyond that, approximately three quarters (71.30%) of migrant women agreed that men should also receive reproductive health education.

### 3.3. Behavior of Informed Choice among Participants

Condom use was the preferred contraceptive method among nearly one half of participants (48.85%). More than 90% (92.66%) of participants decided their contraceptive methods themselves, and 5.26% of participants experienced side effects when using contraceptives. However, 25.29% of them went to public hospitals, and less than one half (45.88%) did not handle side effects. Few participants (10.12%) reported that they received RHS. Additionally, significant differences in the rates in induced abortions occurring at least once were found between Beijing, Shanghai and Chongqing (χ^2^ = 69.93, *p* < 0.0001). After adjusting the region effect, the rate of induced abortions occurring at least once in laborers (35.19%) was significantly higher than that in the other occupations (χ^2^ = 10.75, *p* = 0.0132). There was a significant highest rate of induced abortions occurring at least once (36.36%) in the participants who were in the length of less than 3 years of the first immigration up to now (χ^2^ = 20.30, *p* = 0.0001). The purpose of giving birth for the immigration had the significant lower rate (19.23%) of induced abortions occurring at least once comparing to the other purposes(χ^2^ = 13.94, *p* = 0.0030). The rate of induced abortions occurring at least once was significant higher (42.68%) in the participants who had no children than that in those who had one or more children (χ^2^ = 10.05, *p* = 0.0181). The rate of induced abortions occurring at least once in married participants (31.24%) was significantly lower than that in the unmarried ones (χ^2^ = 4.02, *p* = 0.0449). The rate of induced abortions occurring at least once in daughter preference (40.54%) was significantly higher than that in son preference and no sex preference (χ^2^ = 11.44, *p* = 0.0033). Tubal sterilization had the lowest rate of induced abortions occurring at least once (21.60%) in contraceptive methods and there was a notable rate difference of induced abortions in these methods (χ^2^ = 13.85, *p* = 0.0078).

The rate of induced abortions occurring at least once in FPSPs (21.82%) was significantly lower than that in couples/sexual partners and physicians/community health workers (χ^2^ = 9.13, *p* = 0.0104) and the participants who had side effects had the significantly higher rate of induced abortions occurring at least once (45.29%) than those who had no side effect (χ^2^ = 8.97, *p* = 0.0001) (Table 2).

As indicated by ZINB analysis results, the score of natural contraceptive methods, contraceptive methods in use, deciders of contraceptive methods and side effects along with individual characteristics were significant determinants of the induced abortion outcomes (*p* < 0.05). The following results emerged by controlling for the other variables: (1) immigrant women in Beijing and Shanghai were less likely to undergo induced abortions than those in Chongqing; (2) the older an immigrant woman was, the more likely she was to have had an induced abortion; (3) the less education an immigrant women received, the more likely it was she would undergo an induced abortion; (4) immigrant women who were laborers at construction sites or factories were more likely to have induced abortions; (5) immigrant women who got married were less likely to undergo induced abortions than those who had no marriage link; (6) the shorter the length of the period between first immigration to the present was for immigrant women, the less likely it was they would undergo induced abortions; (7) the shorter the period immigrant women stayed in the city per year, the more likely they were to have induced abortions; (8) the immigrant women who had one child were less likely to have an induced abortion than those who had no children.

The informed choice yielded insight into the association between contraceptive patterns and induced abortions. As expected, the informed choice was found to be substantial. The higher the score of natural contraceptive methods was, the more frequently the induced abortions occurred. Immigrant women who adopted tubal sterilization were less likely to have induced abortions than those who used IUDs, condoms or pills. If FPSPs decided the contraceptive methods for the participants, it was more likely to reduce the occurrence of induced abortions. Additionally, the induced abortions were more likely to happen when side effects occurred. Furthermore, the interactions of number of children, deciders of contraceptive methods and side effects, respectively, combining contraceptive methods were analyzed. As demonstrated by the results, when the participants with one child employed condoms or the participants who had their contraceptive methods decided by FPSPs applied IUDs, the occurrence of induced abortions was more likely to be reduced among them. The participants who suffered side effects using pills, were more likely to have had induced abortions, with an incidence rate ratio (IRR) of 3.42 (Table 3 and Table 4).

## 4. Discussion

The current study demonstrated the annual induced abortion incidence rate of 0.46 among the reproductive-age immigrant women. Because of the different study populations, the induced abortion incidence was different [10,11,12,13] as well. Only one report in China Daily in 2011 mentioned that 70% of the total of 50,000 induced abortions corresponded to immigrant women in a 2008 Beijing survey [14].

In this study, the knowledge of contraceptive methods documented among participants was quite poor. Although the characteristic varieties of the samples from the other studies may confound the findings, the scores of contraceptive method knowledge in this study were supported by previous studies conducted in China [15,16,17,18,19], and in Guatemala [20]. Nevertheless, the scores in the present study were much lower when compared with the scores from the early studies carried out among Chinese women in Hong Kong and Taiwan [21,22]. This study not only analyzed the level of contraception knowledge but also measured its impact on induced abortions. As indicated by the findings, the score of natural contraceptive methods was the lowest among the three methods (*p* < 0.0001). Furthermore, the ZINB result demonstrated that natural contraceptive methods had a weak impact on the occurrence of induced abortion (IRR = 1.004, CI (1.002, 1.01). However, the short-term and long-term contraceptive methods were not significant (*p* > 0.05). As displayed by a similar study, there was no significant difference in knowledge about contraceptives and attitudes toward contraceptives between cases and controls [23]. Another study gave a contrary result whereby greater contraceptive knowledge was associated with the likelihood of not becoming pregnant [22]. First of all, this study was based on a profile that was different from the above two studies. In other words, the cultural backgrounds and study populations were different. Secondly, the level of contraceptive knowledge among the participants in the above studies was high, while the level was relatively low in this study, especially concerning natural contraceptive knowledge. Thirdly, in this study, three types of contraceptive method scores were analyzed separately. Through this means, more detailed information could be extracted (the effect of natural contraceptive methods was observed directly). Nonetheless, only the general cognition of contraceptive knowledge (contraceptive methods were just a part of it) was analyzed in the above two studies. With these results, the low-level knowing of contraceptive methods should be taken seriously. Natural contraceptive methods, including withdraw and rhythm methods, slightly favor the occurrence of induced abortions, indicating that the necessary skills (ejaculation time or safe period calculation) were difficult to control when compared with the use of long-term and other short-term contraceptives. Even grasping this knowledge and usage precisely, the unexpected outcomes still occurred. Even though the information sources were not included in this study, young women preferred to obtain sexual information from peers or the Internet rather than from professional channels. In this regard, the danger of passing on misinformation about sex and contraceptive use among the population should be highlighted [16].

According to the findings, most of participants considered couples or both sides of partners as having the predominant role to decide contraceptive methods. This is consistent with the previous studies [24,25]. For one thing, it indicated that the empowerment of women is improved greatly with the development of informed choice. With the decrease of age, the proportion of selecting contraceptive methods freely by women themselves or both their husbands/partners was increasing, which revealed the changes of women’s initiative and power of decision-making on that subject [26]. On the other hand, it could be found that the proportion of participants deciding contraceptive methods by FPSPs was quite low indeed, implying that in the cities, the service provided by FPSPs is no longer critical. Though higher standards for FPSPs are set by National Health and Family Planning Commission of the People’s Republic of China, more flexible access to information about contraceptive methods has been diluting the guidance and promotion effects of FPSPs concerning contraceptive methods. As a result, it intangibly increases the likelihood of the induced abortions. Out of the total number of participates, around 70% of them agreed that the men should take more contraceptive or reproductive health courses, highlighting that a key population—males—has been neglected in the contraceptive or reproductive health areas of China. A study conducted in Chongqing [27] demonstrated that the reproductive health education mainly targeted at women traditionally in China. At all levels (from national to local), there were professional reproductive health institutions for women. Up to now, however, there have just been a few contraceptive or reproductive health campaigns for men in Chongqing. Thus, in a bid to decrease the induced abortions among participants from the perspective of men by establishing correct contraceptive views and reinforcing informed choice knowledge in men, it is essential to set up man-related institutions and launch man-targeted contraceptive or reproductive health activities.

The priority factors of selecting contraceptives were effectiveness, followed by convenience. Hence, condoms were the first choice of participants, which was the same as the results reported in the previous studies [28,29]. This indicates that the implementation of informed choice and HIV/STD prevention programs, accompanied with the improvement of educational level and reinforcement of health and self-protection awareness among immigrant population, plays a significant role in increasing the likelihood of condom use. As informed choice moves along non-differentially, immigrant women not only expect more ideal methods but hope to obtain adequate access to them. Considering the contraceptive effectiveness, the tubal sterilization is undoubtedly the best one. IUD and tubal sterilization were the most frequently utilized methods between the 1970s and 1990s. Furthermore, the authorities have promoted long-term medical advice guiding contraceptive methods and applied them as the policy of controlling birth levels due to their effectiveness and safety. Meanwhile, the prevalence of tubal sterilization is decreasing steeply following the reform of family planning policy, implementation of informed choice as well as the awareness of women’s rights. However, new sterilization technology may change people’s opinions about sterilization. To be specific, Vasalgel is being developed as a long-acting and non-hormonal contraceptive with one significant advantage: it is likely to be more reversible for men. Hysteroscopic sterilization, a new and less expensive method of plugging the fallopian tubes to prevent conception featured with “no surgical incision and quicker recovery”, has been put into clinical application. Undoubtedly, these new methods will invigorate the informed choice and make more choices available [30,31].

Two intriguing findings, which reduced the number of induced abortions in this study, were the two joint effects, namely the interaction of having one child and condom use as well as the interaction of family planning providers and IUD introduction. The former indicates that as China’s 35-year-old one-child policy is abolished to allow the couples to have a second child, women in China preferred to decide the number and spacing of their children freely and responsibly. Also, they have the freedom to choose and use a safe and acceptable protection method so as to avoid the unplanned pregnancy [32]. This is the centralized reflection of reproductive women’s rights in China with regard to informed choice. The latter highlights two points, one of which is the inertia of policies. According to the history of family planning in China, sterilization and IUD were the leading adopted methods as discussed above. Family planning providers, as the only executants of family planning policies, made great efforts to put IUD into practice either mandatorily or voluntarily. Although the relative policies are being loosened, the policy impact on IUD use will exist for a long time. The other is the gender effect. The earlier survey showed a relative increase in copper IUD use in female physicians when compared with in the general population [33]. Therefore, it could be inferred that the induced abortions were reduced by using policy means. Notably, contraception failures are more likely to rise when more than five types of IUD are provided. This is mainly due to the inadequate access to training FPSPs and the fact they cannot guarantee the correct use [34]. The third interaction of pills with side effects increased the occurrence of induced abortions. As reported in *The Telegraph*, pills can increase the chance of gaining weight, developing blood clots, Crohn’s disease, glaucoma, etc. However, there is still a lack of scientific evidence for this, meaning that more risk factors such as characteristics, lifestyle or medical conditions should be considered [35]. This phenomenon is associated with the inadequacy of primary care doctors’ expertise in hormonal contraception as well as reproductive women’s poor adherence to the instructions for the proper use of pills [35,36]. Many of them are afraid of the side effects of pills, and this can lead to discontinuation and inconsistent use of both methods [36]. Additionally, compared with the reproductive women who choose contraceptive methods aggressively, those who choose methods passively will be more likely to suffer from side effects [35].

The findings of several studies [37,38,39] are similar to those of this study. Namely, there were notable rate differences of induced abortions in different contextual and informed choice factors. In the ZINB model, region, age, educational attainment, occupation, immigration length and marital status were related to the induced abortions. These findings were supported by several studies [40,41,42]. Compared with the eastern region of China, the reproductive health services in the mid-west region feature weaker foundations and uneven development [26]. Meanwhile, unmet contraception needs still exist. Beyond that, the older participants were more likely to have had induced abortions, implying that they were more sexually and reproductively active. Furthermore, the participants with higher educational level and more intellectual work are more likely to have strong awareness of self-protection, contraception and the ability to acquire reproductive health knowledge [41]. Immigration length determines the impact degree on the awareness of contraception among reproductive-age migrant women in the cities. Marital status can be a crucial factor that is associated with the induced abortions. Being married was a protective factor against induced abortions in this study. In the early studies, the similar finding has reported that the induced abortion rate of the unmarried women is higher than that of married women [43,44,45,46,47].

### Limitations

There are several limitations in this study. First of all, the reasons of contraception failure and the effect of emergency contraception are immeasurable, although the previous studies have disputed such reasons about whether the widespread use of emergency contraception could have reduced the abortion rates [48,49,50,51]. Secondly, due to the private and sensitive nature of induced abortions and sexual behavior, the rate of induced abortions could have possibly been underestimated. Thirdly, the nature of cross-sectional will impede the establishment of causal association. Fourthly, recall bias might occur.

## 5. Conclusions

The current study documented a moderately high rate of induced abortions among reproductive-age immigrant women in China. Through the analysis on K.A.P of the population, the findings demonstrated that the knowledge on contraceptive methods was quite poor. Additionally, the autonomous capability to select these methods was enhanced and the pattern of applying long-term methods was shifted to short-term methods among them. More importantly, by independently examining the effect of informed choice and related interaction effects, the way through which the combination of informed choice and fertility needs, family planning providers and side effects influenced the induced abortion numbers among reproductive-age immigrant women has been revealed. To our knowledge, a paucity of study is carried out to examine the effects of contraceptive methods, specifically the interaction effects of these methods and related factors on induced abortions in China. Thus, there is a positive action of curbing unnecessary induced abortions targeting reproductive-age immigrant women by effectively improving the informed choice policies.

## Figures and Tables

**Table 1:** Socio-demographic characteristics of the study subjects (*N* = 3230).

Characteristic	*N*	%
Age		
20–29	932	28.85
30–39	1389	43.00
40–49	909	28.14
Educational attainment		
Elementary school or lower	317	9.81
Junior high school	1312	40.62
High school	855	26.47
College or higher	746	23.10
Occupation		
Laborer	574	17.77
White-collar worker	460	14.24
Service worker	1688	52.26
Other (unemployed or self-employed)	508	15.73
Marital status		
Married	2993	92.66
Unmarried	237	7.34
Family per capita monthly income (yuan)		
<1000	179	5.54
1000–2999	826	25.57
3000–4999	1272	39.38
≥5000	953	29.50
Registered residence status		
Rural	2131	65.98
Urban	1099	34.02
Length of the first immigration up to now (year)		
<3	220	6.81
3–6	687	21.27
7–10	834	25.82
≥11	1489	46.10
Length of stay in city per year (month)		
<7	103	3.19
7–9	70	2.17
≥10	3057	94.64
Purpose for immigration		
Work	2305	71.36
Marriage	785	24.30
Giving birth	52	1.61
Other (business/learning skills)	88	2.72
Whether having medical insurance or not in city		
Yes	1718	53.19
No	1512	46.81
Number of children		
0	154	4.77
1	2040	63.16
2	909	28.14
≥3	127	3.93
Sex preference		
Son preference	339	10.50
No sex preference	2595	80.34
Daughter preference	296	9.16

**Table 2:** Distribution of induced abortions in different contextual and reproductive factors (*N* = 3230).

Variable	Induced Abortions				*χ*^2^^△^	*p-*Value
	0		≥1			
	*N*	%	*N*	%		
Region					69.93	0.000
Beijing	766	69.45	337	30.55		
Shanghai	1055	72.86	393	27.14		
Chongqing	373	54.93	306	45.07		
Occupation *					10.75	0.0132
Laborer	372	64.81	202	35.19		
White-collar worker	323	70.22	137	29.78		
Service worker	1142	67.65	546	32.35		
Other (unemployed or self-employed)	357	70.28	151	29.72		
Length of the first immigration up to now (year) *					20.30	0.0001
<3	140	63.64	80	36.36		
3–6	486	70.74	201	29.26		
7–10	597	71.58	237	28.42		
≥11	971	65.21	518	34.79		
Purpose for immigration *					13.94	0.0030
Work	1576	68.37	729	31.63		
Marriage	517	65.86	268	34.14		
Giving birth	42	80.77	10	19.23		
Other (business/learning skills)	59	67.05	29	32.95		
Number of children *					10.05	0.0181
0	88	57.14	66	42.86		
1	1389	68.09	651	31.91		
2	638	70.19	271	29.81		
≥3	79	62.20	48	37.80		
Marital status *					4.02	0.0449
Married	2058	68.76	935	31.24		
Unmarried	136	57.38	101	42.62		
Sex preference *					11.44	0.0033
Son preference	248	73.16	91	26.84		
No sex preference	1770	68.21	825	31.79		
Daughter preference	176	59.46	120	40.54		
Contraceptive methods *						
IUD	787	67.09	386	32.91	13.85	0.0078
Condom	1066	67.55	512	32.45		
Pill	81	63.78	46	36.22		
Tubal sterilization	225	78.40	62	21.60		
Other	35	53.85	30	46.15		
Deciders of contraceptive methods *						
Couples/sexual partners	2024	67.62	969	32.38	9.13	0.0104
FPSPs	129	78.18	36	21.82		
Physicians/community health workers	41	56.94	31	43.06		
Whether side effect occurred? *						
Yes	93	54.71	77	45.29	8.97	0.0001
No	2101	68.66	959	31.34		

^△^ was the CMH Chi-square test. * adjusted the region effect.

**Table 3:** ZINB analysis of informed choice correlated with induced abortions among immigrant women.

Variable	Reference	Estimate	Standard Error	95% CI	χ^2^	*p-*Value
Region						
Beijing	Chongqing	−0.36	0.11	(−0.58, −0.14)	10.55	0.0012
Shanghai		−0.55	0.10	(−0.75, −0.35)	29.19	0.0000
Age						
30–39	20–29	0.18	0.09	(0.0068, 0.35)	4.15	0.0416
≥40		0.37	0.10	(0.17, 0.56)	13.34	0.0003
Educational attainment						
Elementary school or lower	College or higher	0.31	0.15	(0.0081, 0.61)	4.05	0.0442
Occupation						
Labor	White-collar worker	0.34	0.14	(0.06, 0.62)	5.80	0.0160
Marital status						
Married	Unmarried	−0.31	0.15	(−0.60, −0.02)	4.33	0.0374
Length of the first immigration up to now (year)						
3–6	≥11	−0.33	0.09	(−0.51, −0.15)	13.29	0.0003
Length of stay in city per year (month)						
<7	≥10	0.54	0.22	(0.10, 0.98)	5.80	0.0161
Number of children						
1	0	−0.30	0.13	(−0.56, −0.03)	4.79	0.0286
Score of natural contraceptive methods		0.0035	0.0010	(0.0015, 0.01)	11.41	0.0007
Contraceptive methods						
IUD	Tubal sterilization	0.33	0.13	(0.07, 0.58)	6.09	0.0136
Condom		0.37	0.13	(0.11, 0.64)	7.56	0.0060
Deciders of contraceptive methods						
FPSPs	Couples/sexual partners	−0.45	0.17	(−0.79, −0.12)	7.02	0.0080
Whether or not side effects occurred						
Yes	No	0.36	0.12	(0.13, 0.59)	9.29	0.0023

**Table 4:** ZINB analysis of interaction of informed choice correlated with induced abortions among immigrant women.

	Estimate	Standard Error	95% CI	χ^2^	*p*-Value
One child vs. zero children using condoms	−0.40	0.15	(−0.70, −0.11)	7.16	0.0075
Having side effects vs. not having side effects using pills	1.23	0.40	(0.45, 2.01)	9.54	0.0020
FPSPs vs. Couples/sexual partners using IUD	−0.57	0.22	(−0.99, −0.14)	6.85	0.0089

Note: the references of condoms, pills and IUD were all tubal sterilization. The analysis was controlled by social-demographic, obstetric characteristics, scores of contraceptive methods, contraceptive methods, deciders of contraceptive methods and the fact whether or not the side effects occurred.

## Abbreviations

The following abbreviations are used in this manuscript:

IQR	Inter-Quartile Range
FPSP	Family planning service provider
IUD	Intrauterine device
K.A.P	Knowledge, attitude and practice
RHS	Reproductive health education and service
NB	Negative binomial
LM	Lagrange multiplier
ZINB	Zero-inflated negative binomial
IRR	Incidence rate ratio
